# The Medical Colleges of the City—Their Closing Exercises

**Published:** 1888-04

**Authors:** 


### THE MEDICAL COLLEGES OF THE CITY—THEIR CLOSING EXERCISES.

### ATLANTA MEDICAL COLLEGE.

The closing exercises of the Atlanta Medical College at DeGive's Opera House on the evening of March 1st were exceedingly pleasant and entertaining. The weather was auspicious for the event, and it was a large crowd that wended its way along the streets of the "Gate City" to the 30th annual commencement of the college, and when the curtains rose at eight o'clock, the faculty and friends on the stage faced one of the largest and most cultured audiences that ever graced the building at one of the annual commencements. Wurm's orchestra furnished the music for the occasion and added much to the enjoyment of the evening.

After a most impressive prayer by Rev. G. B. Strickler, D. D., of Atlanta, the Proctor, Dr. W. S. Kendrick, made his report to the Board of Trustees, stating that out of a large attendance during the winter of 1887 and 1888, there were sixty-four in the senior class. Of these fifty-six applied, and fifty-four having passed a satisfactory examination are presented for the degree of Doctor of Medicine.

Dr. H. H. Tucker, President of the Board of Trustees, in his usual happy manner and accomplished style, conferred the degrees upon the following gentlemen: W. H. Bell, N. V. Boddie, J. W. Bramlett, J. P. Brank, T. J. Bridges, M. S. Brown, C. R. Buchanan, L. K. Burruss, B. N. Bussey, W. E. Butner, G. G. O. Chapman, A. J. Christopher, J. D. M. Coleman, W. W. Cornog, W. R. Dendy, A. P. English, T. H. P. Fincher, W. L. Gilbert, W. J. Green, T. M. Greenwood, W. B. Hamby, W. F. Hammack, G. T. Hays, L. F. Henley, P. W. Hoyle, F. M. Hubbard, W. C. Humphries, G. M. Hunt, J. H. Hunter, A. S. Johnson, M. G. Leak, J. G. McAfee, A. B. McRae, O. F. Moran, G. F. Mosely, W. J. Pitman, E. G. Poole, H. B. Potts, F. M. Reeves, C. C. L. Rudicil, B. F. Shamblin, J. L. Shamblin, W. F. Sibbett, J. Y. H. Smith, R. B. Snead, W. L. Story, J. H. Stroud, P. G. Trent, G. C. Trimble, D. H. Vaughan, D. W. Waggoner, J. D. Watkins, I. E. Webb, G. W. Woodliff.

After the diplomas were awarded, Dr. D. W. Waggoner, the valedictorian of the class, delivered an elegant address very suitable to the occasion. But it was left for Rev. Dr. Isaac S. Hopkins, President of Emory College and the orator of the evening, to hold the quiet, earnest attention of the audience, with an address (see p. 65), noted for its conciseness, its adaptability to the occasion, its brilliant though practical thoughts and the ease and elegance with which it was delivered. This was followed by a short, though chaste and beautiful speech by Col. T. P. Westmoreland, of this city, at the conclusion of which he delivered the following prizes:

Faculty prizes—1st honor, W. W. Cornog; 2d honor, J. H. Hunter and F. M. Hubbard; 3d honor, L. F. Henley.

Of the individual prizes of the faculty, Dr. Todd's was awarded to R. B. Snead, Dr. Hardon's to I. E. Webb, Dr. Westmoreland's to J. W. Bramlett, Dr. Calhoun's to J. D. Watkins.

The Proctor reported that the class had been specially noted for its good conduct and morality and contained some of the best men that had ever applied for graduation. After benediction by Rev. T. R. Kendall, M. D., of Macon, the large audience filed out into the open streets and the faculty retired from the stage well pleased with the exercises and with an abiding confidence in the flattering prospects of the future of the institution.

### SOUTHERN MEDICAL COLLEGE.

The ninth annual commencement of the Southern Medical College occurred at DeGive's Opera House on the 29th of February. The exercises were opened with prayer by Rev. John Jones, D. D. The Dean, Dr. Wm. Perrin Nicolson, read his report to the Board of Trustees. He stated the attendance of students in the medical department, session 1887–8, ninety-two, forty of whom were in the senior class; of these thirty-two having passed a successful examination were entitled to the degree of Doctor of Medicine.

Dr. Thos. S. Powell, President of the Board of Trustees, in his most impressive manner, conferred the degree of Doctor of Medicine upon the following gentlemen:

J. F. Beck, B. B. Bell, J. M. Bosworth, J. D. Bradley, G. M. Burnett, C. D. Coker, A. B. Eaton, S. J. Gay, G. W. Hill, Jr., Romeo Hicks, V. H. Hicks, C. H. Holland, J. N.

Statum, A. R. Stephens, J. W. Thomas, C. L. McCaul, M. M. McGehee, A. A. McNeill, D. B. Moore, L. W. Pitchford, J. J. Poore, J. B. Sanders, J. R. Sewell, J. M. Spence, M. D. Jamerson, David Lindlay, J. E. Tucker, W. J. Westbrook, J. F. Williams, Moses Yarbrough, W. J. Tipton, B. M. Trentham.

A. B. Eaton delivered the valedictory to graduates of the medical department.

Dr. L. D. Carpenter, Dean of the Dental Department, which had been organized since the close of the last session, reported twenty-six students in attendance and six graduates, five of whom have attended a full course in the Medical Department.

For this department J. A. Willis was valedictorian.

The annual orator, Hon. J. T. Nisbet, was introduced and delivered an eloquent address.

The prizes were then delivered to the successful candidates.

Faculty prize, first honor—B. B. Bell, of Georgia.

Faculty prize, second honor—A. P. McNeill, of South Carolina.

Obstetrics—Prof. Powell, Gold Medal—J. D. Bradley, of Georgia.

Practice of Medicine—Prof. Bizzell, Gold Medal, S. J. Gay, of Alabama.

Materia Medica—Prof. Roy, Thermometer and Hypodermic Syringe—J. N. Spence, Jr., of Georgia.

Eye, Ear and Throat—Prof. Hobbs, Ophthalmoscope—G. W. Hill, Jr., of Georgia.

Physiology—Prof. Word, Pocket Case Medicines—A. R. Stephens, of Alabama.

Anatomy—Prof. Nicolson, Pocket Case Instruments—A. B. Eaton, of Tennessee.

Surgery—Prof. Gaston, Pocket Case Instruments—W. J. Tipton, of Alabama.

Chemistry—Prof. Burns, Urinary Analysis Set—C. H. Holland, of Georgia.

Major M. C. Kiser, one of the trustees, generously offered a prize to the student writing the best thesis. This prize was awarded to A. B. Eaton, of Tennessee.

---

### ITEMS.

ON the 29th of February Dr. Miller B. Hutchins, of this city, was married to Miss Addie Davis, of Savannah. THE JOURNAL extends its best wishes to the happy couple.

CONGRATULATIONS.—Dr. S. W. Pryor (A. M. C., 1887), of Union county, S. C., was married, February 14, 1887, to Miss Maggie Tinsley, of the same place.

THE Medical Association of Alabama will meet in Montgomery April 10th and continue in session four days. An interesting programme has been arranged, and the usual large attendance is anticipated. The work of this well-organized society speaks for itself, and words of praise from us are superfluous.

ON the first instant Dr. F. W. McRae was united in marriage to Miss Fannie Collier—both of this city. The ceremony was performed at the First Methodist Church by Rev Dr. Morrison, and was witnessed by a large gathering of members of the profession and other invited friends. THE JOURNAL extends to Dr. McRae and his bride its warmest congratulations and wishes him a degree of happiness proportionate to his deserts. We could not wish more.

A NEW FORM OF INFECTIOUS PNEUMONIA.—At the recent Medical Congress in Italy, Professor Cantani presented a communication on a new form of infectious pneumonia which had been observed by him. The clinical history showed broncho-pneumonia, which had been preceded by a diffuse bronchitis with remittent and very pronounced fever, considerable emaciation and great enlargement of the spleen. The disease was contagious and was a primary affection of the bronchi, which extended downward through the lung, and sometimes over the pleura and upwards along the trachea and even to the larynx and pharynx. Bacteriological examination revealed the presence of numerous diplococci, and especially streptococci, similar to those found in erysipelas. The pure cultures did not, however, produce erysipelas when injected subcutaneously. When they were injected under the skin of a rabbit's ear, only swelling and reddening at the site of puncture were produced. All the cases ran a favorable course.—*British Medical Journal.*

FIXING FOR PHYSICIANS.—Pursuant to the call in the *Tribune* of Sunday, the physicians of the city met last night at the city hall to arrange for the entertainment of the Georgia Medical Association, which convenes in Rome on April 18th.

Dr. Robert Battey is chairman of the Committee of Arrangements, and the following committees were appointed:

On Hotels—Dr. T. M. Holmes.

On Barbecue—Dr. G. W. Holmes, Dr. W. D. Hoyt, Dr. H. H. Battey.

Free Transportation and Boat Excursion—Dr. J. B. S. Holmes.

On Place of Meeting—Dr. J. W. Janes and Dr. H. H. Bradley.

On Seeing the Mayor and Extending Invitations to Ladies to Assist in Entertaining—Dr. Robert Battey.

Finance Committee—Dr. J. B. S. Holmes, Dr. H. H. Battey and Dr. T. M. Holmes.

To see Dr. Gwaltney and Professor Caldwell about Concerts and Art Displays—Dr. J. W. Janes.

Private Entertainments—Dr. Robert Battey and Dr. J. B. S. Holmes.

On Decorations—Dr. T. M. Holmes.

On Badges—Dr. J. W. Janes and Dr. M. Fink.—*Rome Tribune.*

SUCCESSFUL EXCISION OF A TUMOR OF THE SPINAL CORD.—Surgery is a science, or perhaps we should say a fine art, which will tolerate no limit to its domain. It has of late taken up the invasion of the brain in earnest; it has just made its first successful dash at a tumor in the spinal cord. Last Tuesday evening, before the meeting of the Medical and Chirurgical Society, a private patient of Dr. Gowers and Mr. Victor Horsley very generously allowed the fellows and visitors of that society the opportunity of seeing all that had been done for the improvement of his condition. He had spent about three years in severe pain, which was most intense just below and inside the angle of the left scapula, and was accompanied by absolute loss of motion and sensation of the body and limbs below that level. The upper border of the anæsthesia was distinctly in the region of the fifth intercostal nerve on the left side; on the right it was less accurately defined, but did not extend higher. All the symptoms agreed with those of tumors of the spinal cord, and the intense pain afforded ample justification for making an attempt to excise the tumor. Mr. Victor Horsley accordingly removed the spines and parts of the laminæ of the fifth and fourth dorsal vertebræ; but not until the third vertebra had been similarly treated did the tumor come into sight. It was a small, oval myxoma compressing and making a deep impression on the left side of the spinal cord below the third vertebra. It was easily shelled out, and under careful antiseptic treatment the temperature did not rise more than 1° F. The wound healed rapidly, except at the uppermost point, where a drain had been left in by which a little cerebro-spinal fluid flowed away very slowly. For three or four weeks the former acute pain did not lessen, and even at times seemed more agonizing, but after that it gradually and intermittently decreased, and now, after seven months, is entirely gone; the sensation and motion of the body and legs are almost completely restored. This is, we believe, the first time that such an operation has been attempted, and we must most heartily congratulate both the patient and his advisers on the triumphant character of its success. However far, and however quickly surgery may advance, it will long be a memorable day when it gained its first victory on so new a field and over so formidable an enemy.—*British Medical Journal.*

THE thirty-ninth annual session of the American Medical Association will be held in Cincinnati, Ohio, on Tuesday, Wednesday, Thursday and Friday, May 8, 9, 10 and 11, commencing on Tuesday at 11 a. m.

"The delegates shall receive their appointment from permanently organized State medical societies, and such county and district medical societies *as are recognized by representation in their respective State societies*, and from the medical department of the army and navy and the marine hospital service of the United States.

"Each State, county and district medical society entitled to representation shall have the privilege of sending to the Association one delegate for every ten of its regular resident members, and one for every additional fraction of more than half that number; *Provided*, however, that the number of delegates for any particular State, territory, county, city or town shall not exceed the ratio of one in ten of the resident physicians who may have signed the code of ethics of the Association."

SECTIONS.

Practice of Medicine, Materia Medica and Physiology—Dr.

*———, Chairman ; Dr. N. S. Davis, Jr., 65 Randolph street, Chicago, Ill., Secretary.

Obstetrics and Diseases of Women and Children—Dr. Eli Van De Warker, 45 Montgomery street, Syracuse, N. Y., Chairman ; Dr. E. W. Cushing, 1 Hotel Pelham. Boston, Mass., Secretary.

Surgery and Anatomy—Dr. Donald McLean, 72 Lafayette Avenue, Detroit, Mich., Chairman ; Dr. B. A. Watson, 124 York street, Jersey City, N. J., Secretary.

State Medicine—Dr. H. B. Baker, Lansing, Mich., Chairman ; Dr. S. T. Armstrong, U. S. M. Hospital Service, Secretary.

Ophthalmology, Otology and Laryngology—Dr. F. C. Hotz, 181 Clark street, Chicago, Ill., Chairman ; Dr. Edward Jackson, 215 S. 17th street, Philadelphia, Pa., Secretary.

Diseases of Children—Dr. F. E. Waxham, 3449 Indiana Ave., Chicago, Ill., Chairman ; Dr. W. B. Lawrence, Batesville, Ark., Secretary.

Oral and Dental Surgery—Dr. J. Taft, Cincinnati, Ohio, Chairman ; Dr. E. S. Talbot, 125 State street, Chicago, Ill, Secretary.

Medical Jurisprudence—Dr. E. M. Reid, 243 N. Fremont street, Baltimore, Md., Chairman ; Dr. C. B. Bell, Suffolk, Mass., Secretary.

Dermatology and Syphilography—Dr. L. D. Bulkley, 4 E. 37th street, New York, Chairman ; Dr. S. F. Dunlap, Danville, Ky., Secretary.

☞A member desiring to read a paper before a section should forward the paper, or its *title* and *length* (not to exceed twenty minutes in reading) to the chairman of the Committee of Arrangements at least one month before the meeting.—*By-laws.*

Committee of Arrangements—W. W. Dawson, Cincinnati, Ohio, Chairman.

WM. B. ATKINSON, M. D.,
*Permanent Secretary.*

*Vacant.

